# Protective Effect of Astaxanthin on Liver Fibrosis through Modulation of TGF-*β*1 Expression and Autophagy

**DOI:** 10.1155/2014/954502

**Published:** 2014-04-17

**Authors:** Miao Shen, Kan Chen, Jie Lu, Ping Cheng, Ling Xu, Weiqi Dai, Fan Wang, Lei He, Yan Zhang, Wang Chengfen, Jingjing Li, Jing Yang, Rong Zhu, Huawei Zhang, Yuanyuan Zheng, Yingqun Zhou, Chuanyong Guo

**Affiliations:** Department of Gastroenterology, Shanghai Tenth People's Hospital, Tongji University of Medicine, Shanghai 200072, China

## Abstract

Liver fibrosis is a common pathway leading to cirrhosis and a worldwide clinical issue. Astaxanthin is a red carotenoid pigment with antioxidant, anticancer, and anti-inflammatory properties. The aim of this study was to investigate the effect of astaxanthin on liver fibrosis and its potential protective mechanisms. Liver fibrosis was induced in a mouse model using CCL4 (intraperitoneal injection, three times a week for 8 weeks), and astaxanthin was administered everyday at three doses (20, 40, and 80 mg/kg). Pathological results indicated that astaxanthin significantly improved the pathological lesions of liver fibrosis. The levels of alanine aminotransferase aspartate aminotransferase and hydroxyproline were also significantly decreased by astaxanthin. The same results were confirmed in bile duct liagtion, (BDL) model. In addition, astaxanthin inhibited hepatic stellate cells (HSCs) activation and formation of extracellular matrix (ECM) by decreasing the expression of NF-*κ*B and TGF-*β*1 and maintaining the balance between MMP2 and TIMP1. In addition, astaxanthin reduced energy production in HSCs by downregulating the level of autophagy. These results were simultaneously confirmed in vivo and in vitro. In conclusion, our study showed that 80 mg/kg astaxanthin had a significant protective effect on liver fibrosis by suppressing multiple profibrogenic factors.

## 1. Introduction

Liver fibrosis is a wound-healing response and can be caused by a multitude of pathological factors, such as hepatitis viruses, metabolic diseases, and alcohol. It is characterized by the excessive deposition of extracellular matrix (ECM), due to an imbalance between ECM synthesis and degradation [1]. The activation of hepatic stellate cells (HSCs) after liver injury is regarded as the most pivotal event underlying hepatic fibrogenesis [2]. During this process, HSCs transform from quiescent HSCs into proliferative and contractile myofibroblast-like cells, along with plentiful generation of collagen type I, one of the principal components of ECM. Activated HSCs express new proteins such as *α*-smooth muscle actin and new receptors such as the platelet-derived growth factor (PDGF) receptor, which can reflect the proliferative status of activated HSCs [3, 4]. There are many types of cytokines and chemokines related to the activation of HSCs. Transforming growth factor (TGF)-*β*1 is considered the most critical cytokine to participate in this process [5, 6]. When liver injury occurs, related cells, such as Kupffer cells and sinusoidal endothelial cells, are activated to release various cytokines, finally resulting in the activation and proliferation of HSCs. In addition, TGF-*β*1 can regulate the expression of matrix metalloproteinases (MMPs) and tissue inhibitor of matrix metalloproteinases (TIMPs). MMPs have the ability to degrade multiple components of basement membrane which play an important role in the formation of ECM. TIMPs have an adverse function. Particularly, MMP2 and TIMP1 are expressed primarily in liver fibrosis [7, 8]. Therefore, TGF-*β*1 is considered a target to inhibit the activation of HSCs and ameliorate liver fibrosis.

It is well known that the activation of HSCs is an essential procedure contributing to liver fibrosis. As previously mentioned, TGF-*β*1 is one of the most important cytokines in this process. However, the energy metabolism of HSCs themselves after activation is also necessary to ensure survival and proliferation. Quiescent HSCs contain many lipid droplets, and when these cells are activated, the lipid droplets decrease and provide energy. Recently, Friedmans reported that autophagy can regulate lipid metabolism in HSCs to promote the activation of HSCs [9]. The same results were also demonstrated by other scientists [10, 11]. Autophagy is a metabolic process where cells degrade their own components under exogenetic stimulation, such as starvation, infection, and hypoxia. There are three types of autophagy: microautophagy, macroautophagy, and chaperone-mediated autophagy [12]. Macroautophagy (hereafter referred to as autophagy) starts with the formation of autophagosomes, which transport cytoplasmic material to lysosomes. The formation of autophagosomes is representative of autophagy. LC3, which is specifically expressed on the membrane of autophagosomes, is used as a marker of autophagy. In addition, beclin-1 is also an important factor in promoting autophagy [13]. Autophagy is a conventional metabolic pathway and is regulated by several signal pathways, such as MAPK, AMPK, and PI3k/AKT [14, 15]. Therefore, it is reasonable that inhibition or downregulation of autophagy in HSCs may attenuate liver fibrosis.

Liver fibrosis is a transient process before the end stage which is cirrhosis and is caused mainly by chronic liver injury. Liver cirrhosis significantly increases the risk of hepatocellular carcinoma, which is a threat to human health [16]. In this long-term advanced disease, liver fibrosis may be a unique process that can be reversed. Hence, it is necessary to identify an effective drug or strategy to ameliorate liver fibrosis to decrease the risk of hepatocellular carcinoma. Astaxanthin is a red carotenoid pigment, which is abundant in marine animals, such as shrimp, salmon, and crab [17, 18]. It has been reported that astaxanthin has powerful antioxidant activity against multiple diseases both in vivo and in vitro [19]. For example, Korea et al confirmed a decade ago that astaxanthin could ameliorate CCL4-induced acute liver injury [20]. Recently, the protective effect of astaxanthin on hepatocellular injury following ischemia/reperfusion was also reported [21]. This not only showed the antioxidant effect of astaxanthin on acute injury, but also astaxanthin exhibited a protective effect on carcinoma and chronic diseases such as fibrosis. Kavitha et al. reported that astaxanthin had the ability to induce intrinsic apoptosis in a hamster model of oral cancer by inhibiting the Erk/MAPK signal pathway [22]. It was previously demonstrated that astaxanthin could attenuate both lung fibrosis and renal fibrosis [23, 24]. However, the effect of astaxanthin on liver fibrosis has not been explored, and it is unclear whether astaxanthin could ameliorate liver fibrosis by regulating the expression of TGF-*β*1 and autophagy. In the present study, we used CCL4 injection and bile duct ligation to induce liver fibrosis in mice and to further investigate the effect of astaxanthin on liver fibrosis and its potential protective mechanisms.

## 2. Materials and Methods

### 2.1. Materials

Astaxanthin was purchased from Sigma Aldrich (St Louis, MO, USA). Carbon tetrachloride (CCL4) was obtained from Sinopharm (China). Polyclonal antibodies against collagen I, beclin-1, LC3, MMP2, *α*-SMA, and Smad3 were purchased from Cell Signaling Technologies (Beverly, MA, USA), the antibody against NF-*κ*B was purchased from Abcam (Cambridge, MA, USA), and antibodies against TGF-*β*1 and PDGFR were purchased from Santa Cruz Biotechnology Inc. (Santa Cruz, CA, USA). Antibodies against TIMP1, lamin A, and Smad2 were purchased from Proteintech (Proteintech, CA, USA).

### 2.2. Animals

Male C57 mice (6–8 weeks old, 22 ± 2 g) were purchased from Shanghai Laboratory Animal Co., Ltd. (SLAC, Shanghai, China). The mice were housed in an air-conditioned room at 24°C with a 12 h dark/light cycle and permitted free access to standard laboratory food and water. All animal experiments were approved by the Animal Care and Use Committee of Shanghai Tongji University. For chronic CCL4-induced liver fibrosis, 2.0 mL/kg of 6% CCL4 (v/v) solution in peanut oil was administered by intraperitoneal injection three times a week (Monday, Wednesday, and Friday) for 8 weeks. Astaxanthin was diluted in peanut oil and was administered orogastrically at daily doses of 20, 40, and 80 mg/kg once per day. A total of 35 mice were prepared and divided into 5 groups including the vehicle group (peanut oil only), CCL4 group, and AST-treated group (three different doses). All animals were sacrificed at the end of the 8-week period, and liver samples and blood were obtained. For bile duct ligation- (BDL-) induced liver fibrosis, 30 mice were averagely divided into three groups (normal group, BDL group, and AST-treated group) and anesthetized with 1.25% Nembutal (Saint Louis, MO, USA). After that, the peritoneal cavity was opened and the common bile duct was double-ligated using 7-0 silk in BDL and AST-treated group. The mice in normal group just opened the peritoneal cavity. Starting on the day of surgery, astaxanthin was administrated orogastrically at daily dose of 80 mg/kg in AST-treated group. All mice were sacrificed at 14 d after surgery and liver samples and blood were acquired.

### 2.3. Biochemical Indicators of Liver Function

Serum was separated from blood which was obtained from all animals. The levels of alanine aminotransferase (ALT) and aspartate aminotransferase (AST) were determined using an automated chemistry analyzer (Olympus AU1000, Japan). The hepatic hydroxyproline content was determined according to the kit instructions (Biocheck, USA).

### 2.4.  Histological Examination

For histological evaluation, all liver samples were fixed in formalin, embedded in paraffin, and 3 *μ*m thick sections were prepared and stained with hematoxylin and eosin (H&E), Masson's trichrome (MT), and sirius red stain (SR). These specimens were observed under a light microscope.

### 2.5. Western Blotting

Liver tissues were dissected and homogenized in RIPA buffer (Kaiji, China). And HCS-T6 and NCTC1469 cells were washed twice with PBS solution and lysed with RIPA buffer. Protein concentrations were measured using the bicinchoninic acid (BCA) protein assay (Kaiji, China). Equal amounts of protein were separated by SDS-PAGE and transferred to PVDF membranes. The membranes were then blocked with 5% milk (dissolved in PBS). One hour later, the membranes were incubated overnight at 4°C with specific antibodies: anti-MMP2 (1 : 500), anti-TIMP1 (1 : 500), anti-*α*-SMA (1 : 500), anti-PDGFb (1 : 500), anticollagen I (1 : 500), anti-TGF-*β*1 (1 : 500), anti-NF-*κ*B (1 :  500), antibeclin-1 (1 : 500), anti-LC3 (1 : 500), anti-Smad2 (1 : 500), anti-Smad3 (1 : 500), antilamin A (1 : 1000), and antiactin (1 : 1000). On the second day, the membranes were washed with PBST and incubated with secondary antibody (goat anti-mouse or goat anti-rabbit, 1 : 2000) for 30 min at 37°C. Finally, the membranes were washed with PBST three times for 5 min each time and the proteins were detected using the Odyssey two-color infrared laser imaging system.

### 2.6. Immunohistochemical Staining

Liver sections (3 *μ*m) were dewaxed and rehydrated regularly and then treated with 3% H_2_O_2_. The sections were then pretreated with a microwave antigen retrieval technique. Then the nonspecific sites were blocked with 10% goat serum for 30 min in room temperature. The specimens were then incubated overnight at 4°C with the following antibodies: anti-*α*-SMA (1 : 500), anti-PDGFb (1 : 500), anticollagen I (1 : 500), anti-TGF-*β*1 (1 : 500), anti-NF-*κ*B (1 : 500), antibeclin-1 (1 : 500), and anti-LC3 (1 : 500). In particular, when stained with NF-*κ*B, the membranes were ruptured with 0.2% triton at room temperature for 30 min before being blocked. On the second day, after incubation with secondary antibody, an antibody binding analysis was performed using a DAB kit. Finally, the slides were counterstained with hematoxylin and observed under a light microscope.

### 2.7. Immunofluorescence

The HSC-T6 cells were washed three times with PBS solution for 1 min. The cells were fixed with 4% paraformaldehyde and washed three times with PBS solution for 5 min again. Nonspecific antigen binding sites were blocked with 5% BSA and then cells were incubated overnight with anti-alpha-SMA antibody (1 : 500) at 4°C. On the second day, cells were washed three times with PBS solution for 5 min and incubated with anti-mouse antibody for 30 min. After that, the nuclei were stained with 2-(4-amidinophenyl)-6-indolecarbamidine dihydrochloride (DAPI) (1 : 1000) for 3 min. All cells were observed under a fluorescence microscopy.

### 2.8. Electron Microscopy

Sections of the liver tissues were fixed with 2% glutaraldehyde buffered with 0.2 mmol/L cacodylate and postfixed in osmium tetroxide before embedding in epoxy resin for electron microscopy. Ultrathin sections were stained with uranyl acetate and lead citrate. The sections were then observed under an electron microscope (FEI Tecnai G2 20 TWIN).

### 2.9. Quantitative Real-Time PCR Analysis

Total RNA was extracted from liver tissues using TRIzol reagent (TIANGEN Biotech, China) as described by the manufacturer. RNA was reverse-transcribed using the Reverse Transcription Kit (TaKaRa Biotechnology, China). To detect the expression of target genes in the liver, quantitative RT-PCR was performed with SYBR Premix EX Taq (TaKaRa Biotechnology, China) using a 7900HT fast real-time PCR system (ABI, CA, USA). Primer sequences are shown in Table 1.

### 2.10. Cell Culture and MTT Assay

NCTC1469 cells and HSC-T6 and RAW264.7 cells were purchased from Chinese Academy of Science Committee Type Culture Collection Cell Bank. NCTC1469 cells were cultured in RPMI-1640 medium. HSC-T6 and RAW264.7 cells were cultured in high glucose Dulbecco's modified Eagle's medium (DMEN-h; Thermo, China) supplemented with 10% fetal bovine serum (Hyclone, South America) and 1% penicillin-streptomycin (Gibco, Canada) in a humidified incubator at 37°C in 5% CO_2_. HSC-T6 cells were plated at a density of 2 × 10^4^ cells/well in 96-well plates. Here, we designed three groups as control group, TGF-*β*1 group, and TGF-*β*1 + AST group. The concentration of TGF-*β*1 was 5 ng/mL, and the concentrations of AST were set as 20, 40, 60, 80, 100, 120, 140, and 160 *μ*m. Cell viability was measured using and MTT assay (Peptide Institute Inc, Osaka, Japan) and a microplate reader at wavelength of 490 nm.

### 2.11. Detection of Apoptosis Using Flow Cytometry

The apoptosis of NCTC1469 cells was administrated using FITC (BD Pharmingen, San Jose, CA, USA). The percentage of early apoptosis and late apoptosis was calculated and analyzed.

### 2.12. Statistical Analysis

The positive areas of MT, SR, and immunohistochemical staining are analyzed by Image-Pro Plus 6.0. IOD (integrated optical density) is used in our research, which can exactly reflect the total protein expression in immunohistochemical staining. And IOD represents the value of density (mean) by area. The Results were expressed as mean ± SD. Statistical comparisons were made using one-way ANOVA with the SPSS 17.0 statistical package. *P* < 0.05 was considered significant.

## 3. Results

### 3.1. Astaxanthin Alleviated Fibrous Changes in the Liver

**Figure 1:**
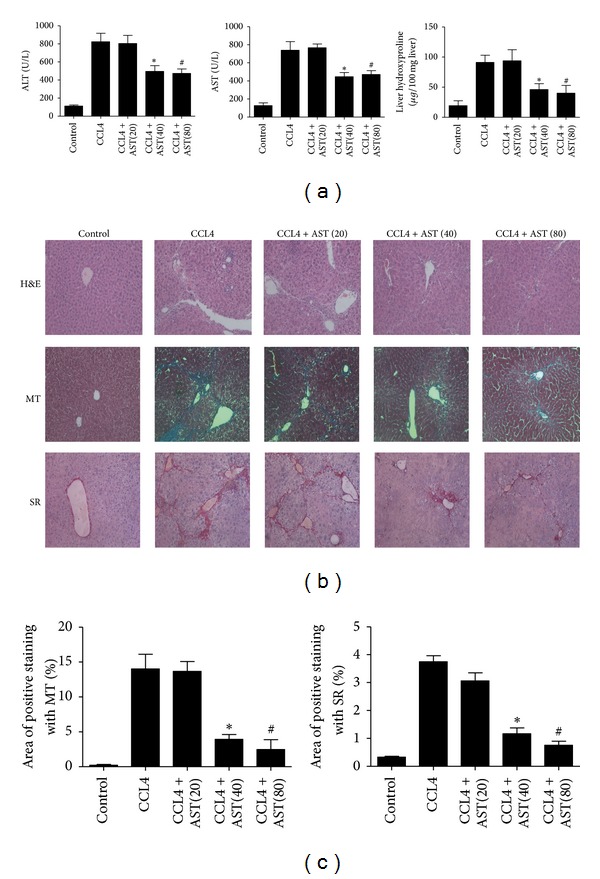
Effect of astaxanthin on CCL4-induced liver fibrosis. (a) Astaxanthin decreased the level of ALT, AST, and hydroxyproline with the doses of 40 mg/kg and 80 mg/kg. Data are expressed as mean ± SD (*n* = 7, *P < 0.05 for CCL4 + AST(40) versus CCL4, ^#^ *P* < 0.05 for CCL4 + AST(80) versus CCL4). (b) Astaxanthin (40 mg/kg and 80 mg/kg) ameliorated pathological change showed by H&E, Masson's trichrome (MT), and sirius red staining (original magnification: ×200). (c) The areas of positive staining with MT and SR were analyzed by Image-pro Plus 6.0. There existed a significant decrease with astaxanthin (40 mg/kg and 80 mg/kg) treatment (*n* = 7, *P < 0.05 for CCL4 + AST(40) versus CCL4, ^#^ *P* < 0.05 for CCL4 + AST(80) versus CCL4).

**Figure 2:**
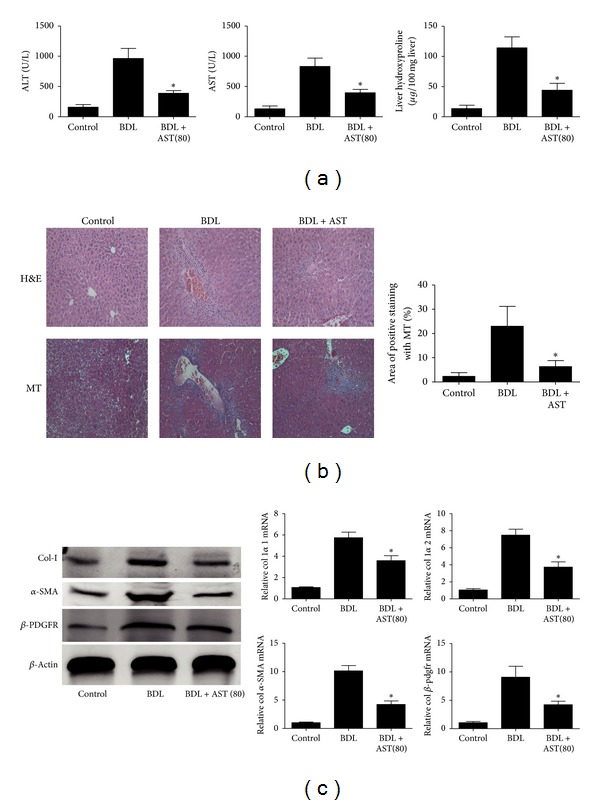
Effect of astaxanthin on BDL-induced liver fibrosis. (a) Astaxanthin decreased the level of ALT, AST, and hydroxyproline with the dose of 80 mg/kg. Data are expressed as mean ± SD (*n* = 10, *P < 0.05 for BDL + AST(80) versus BDL). (b) Astaxanthin (80 mg/kg) ameliorated pathological change showed by H&E and Masson's trichrome (MT) (original magnification: ×200). The areas of positive staining with MT were analyzed by Image-pro Plus 6.0. There existed a significant decrease with astaxanthin (80 mg/kg) treatment (*n* = 10, *P < 0.05 for BDL + AST(80) versus BDL). (c) The analysis of western blotting and real-time PCR showed that astaxanthin obviously decreased the expression of *α*-SMA, *β*-pdfgr, and collagen I with the doses of 80 mg/kg in BDL model (*n* = 3, *P < 0.05 for BDL + AST(80) versus BDL).

To examine the effect of astaxanthin on liver fibrosis, we first determined ALT and AST levels in serum. A significant increase in both ALT and AST was observed in the CCL4-treated group compared to the control group. Serum levels of ALT and AST decreased significantly after astaxanthin treatment (40 mg/kg and 80 mg/kg). However, 20 mg/kg of astaxanthin had no effect (Figure 1(a)). Liver sections were stained with H&E to evaluate liver injury. Severe liver injury and fibrosis were observed in the CCL4-treated group, with necrosis of hepatocytes, damage to liver lobules, and pericellular bridging fibrosis. These changes were markedly reduced in liver sections from the astaxanthin-treated group (40 mg/kg and 80 mg/kg) (Figure 1(b)). Fibrosis is the foremost characteristic of chronic liver injury, with collagen deposits seen in fibrotic tissue. Therefore, we determined the level of hydroxyproline in liver tissue, which reflects total collagen content. Astaxanthin at 40 mg/kg and 80 mg/kg decreased the level of hydroxyproline (Figure 1(a)). We used Masson's trichrome (MT) and sinus red (SR) to stain collagen. Both of these histopathological analyses revealed increased collagen around the portal triad, the lobules were surrounded by bundles of collagen fibers, normal structure was lost, and large fibrous septa were present in the CCL4-treated group (Figure 1(b)). Consistent with ALT and AST analyses and H&E staining, the area and thickness of collagen fiber bundles (stained blue and red, respectively) were decreased following administration of astaxanthin (40 mg/kg and 80 mg/kg). A statistically significant difference in MT and SR-staining was noted between astaxanthin-treated group (40 mg/kg and 80 mg/kg) and CCL4 group, when the staining intensity was analyzed with the software named Image-Pro Plus 6.0 (Figure 1(c)). In addition, astaxanthin (80 mg/kg) was also administrated in BDL model, and results showed that the fibrosis in BDL model was significantly ameliorated by astaxanthin (80 mg/kg) (Figure 2). These results indicated that astaxanthin ameliorated liver fibrosis.

### 3.2. Astaxanthin Inhibited the Activation of HSCs in Liver Fibrosis

**Figure 3:**
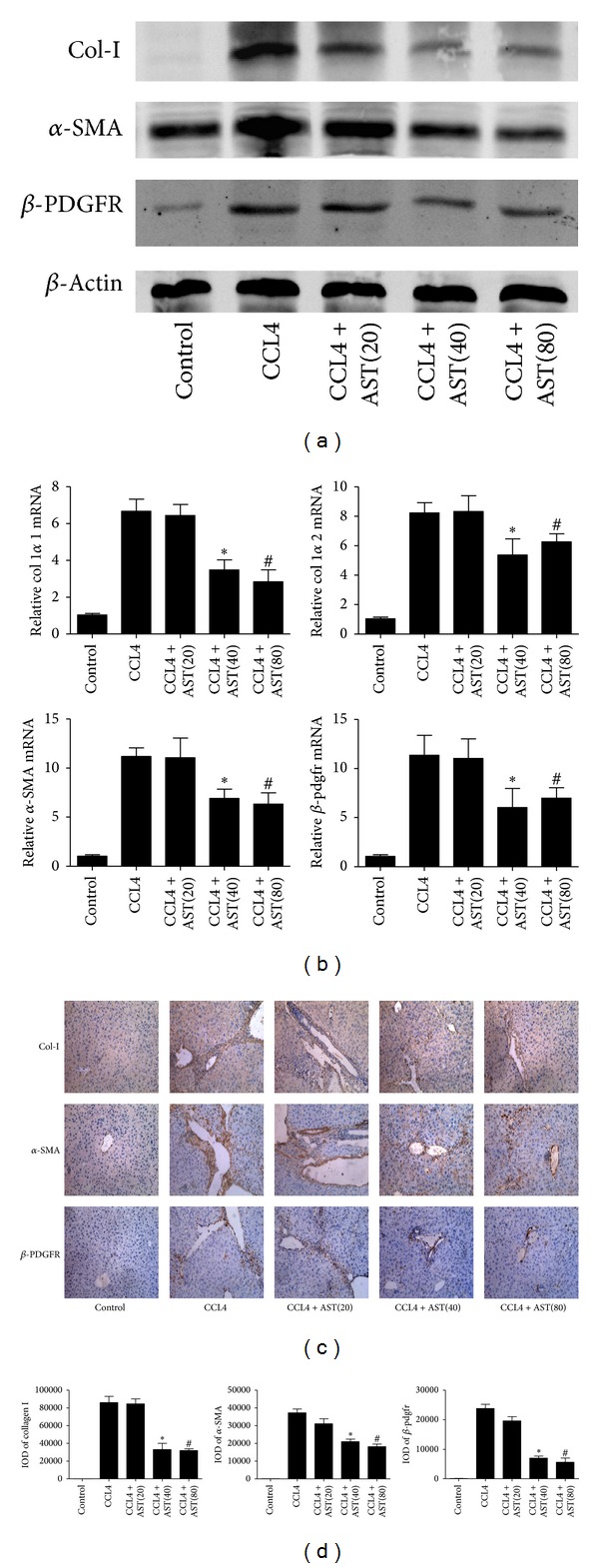
Effect of astaxanthin on the activation of HSCs. (a) The analysis of western blotting showed that astaxanthin obviously decreased the expression of *α*-SMA, *β*-pdfgr, and collagen I with the doses of 40 mg/kg and 80 mg/kg. (b) The mRNA levels of collagen I  *α*1, collagen I  *α*2, *α*-SMA, and *β*-pdgfr were significantly downregulated by astaxanthin (40 mg/kg and 80 mg/kg). Data are expressed as mean ± SD (*n* = 7, *P < 0.05 for CCL4 + AST(40) versus CCL4, ^#^ *P* < 0.05 for CCL4 + AST(80) versus CCL4). (c) The areas of positive cells of *α*-SMA, *β*-pdfgr, and collagen I were diminished by astaxanthin (40 mg/kg and 80 mg/kg), showed by immunohistochemistry staining (original magnification: ×200). (d) The IODs of *α*-SMA, *β*-pdfgr, and collagen I were analyzed by Image-pro Plus 6.0. There existed a significant decrease with astaxanthin (40 mg/kg and 80 mg/kg) treatment (*n* = 7, *P < 0.05 for CCL4 + AST(40) versus CCL4, ^#^ *P* < 0.05 for CCL4 + AST(80) versus CCL4).

Evidence indicates that HSCs play a pivotal role in liver fibrosis, because they are the main matrix-producing cells. Hence, quantitative analysis of HSC activation could predict the progression of liver fibrosis. Following transdifferentiation from quiescent cells to myofibroblast-like cells, *α*-SMA is selectively expressed in HSCs and is the most frequently used marker to indicate HSCs activation. In addition, platelet-derived growth factor (PDGF) is one of the most important cytokines in HSCs activation. Accordingly, *β*-PDFGR would be expressed in HSCs when transformed and may be a marker of HSCs activation. In the present study, we determined the expression of *α*-SMA, *β*-PDFGR, and collagen I to assess the activation status of HSCs in the liver. The results showed that astaxanthin (40 mg/kg and 80 mg/kg) significantly reduced the expression of mRNA encoding collagen I *α*1, collagen I *α*2, *α*-SMA, and *β*-PDGFR and their corresponding proteins (Figures 3(a) and 3(b)). Furthermore, we observed the expression of *α*-SMA, collagen I, and *β*-PDGFR by immunohistochemical staining. Similar to MT and SR staining, the *α*-SMA, collagen I, and *β*-PDGFR positive areas were visibly reduced after astaxanthin administration (40 mg/kg and 80 mg/kg) (Figure 3(c)), analyzed with Image-Pro Plus 6.0. In addition, results showed that astaxanthin (80 mg/kg) could also inhibit the activation of HSCs in BDL model (Figure 2). These results showed that astaxanthin inhibited the activation of HSCs through unknown mechanisms.

### 3.3. Astaxanthin Regulated the Expression of TGF-*β*1, MMP2, and TIMP1 in Liver Fibrosis

**Figure 4:**
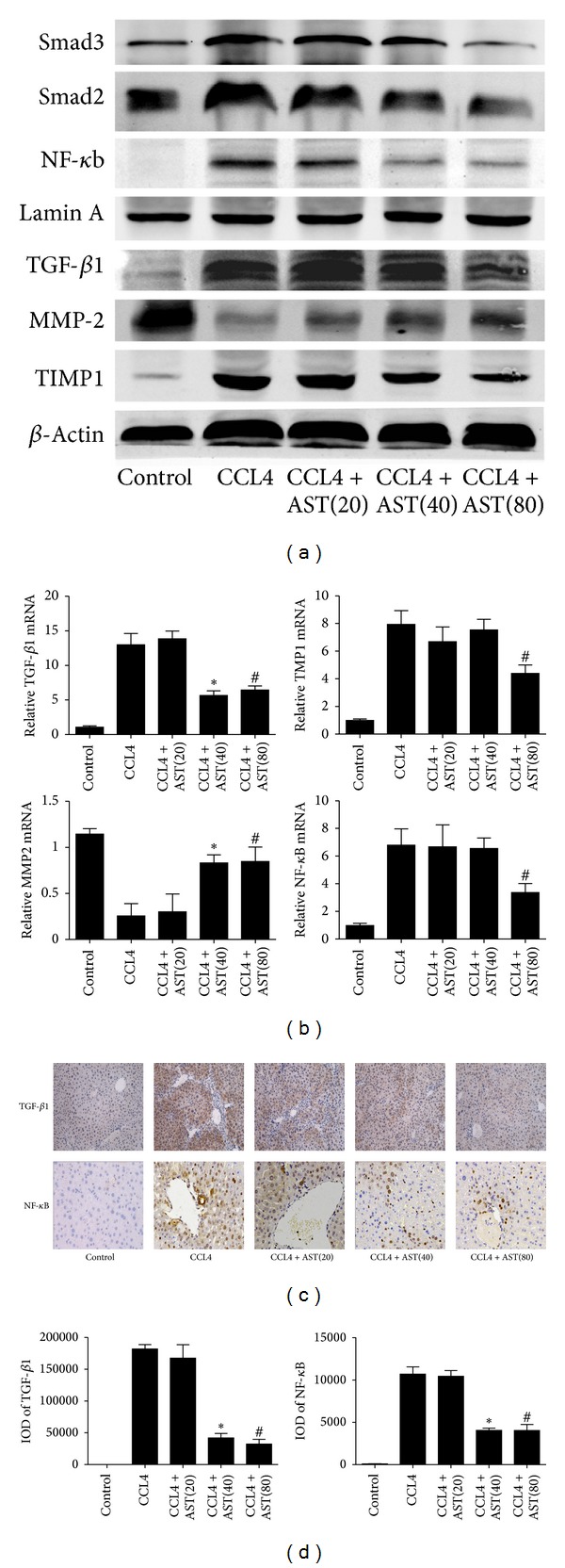
Effects of astaxanthin on expression of TGF-*β*1, MMP2, TIMP1, and NF-*κ*b. (a) The analysis of western blotting showed that astaxanthin decreased the expression of TGF-*β*1, TIMP1, and NF-*κ*b and, contrarily, increased the expression of MMP2 compared to CCL4 group. (b) The mRNA level of TGF-*β*1 was decreased by astaxanthin (40 mg/kg and 80 mg/kg), and the mRNA levels of TIMP1 and NF-*κ*b were decreased with the dose of 80 mg/kg. However, the mRNA levels of MMP2 were increased by astaxanthin (40 mg/kg and 80 mg/kg) compared to CCL4 group. Data are expressed as mean ± SD (*n* = 7, *P < 0.05 for CCL4 + AST(40) versus CCL4, ^#^ *P* < 0.05 for CCL4 + AST(80) versus CCL4). (c) The area of positive cells of TGF-*β*1 was significantly decreased by astaxanthin (40 mg/kg and 80 mg/kg) stained by immunohistochemistry (original magnification: ×200). The expression of NF-*κ*B in nuclei decreased obviously with astaxanthin (40 mg/kg and 80 mg/kg) treatment (original magnification: ×400). (d) The IODs of TGF-*β*1 and NF-*κ*b were analyzed by Image-pro Plus 6.0. There existed a significant decrease with astaxanthin (40 mg/kg and 80 mg/kg) treatment (*n* = 7, *P < 0.05 for CCL4 + AST(40) versus CCL4, ^#^ *P* < 0.05 for CCL4 + AST(80) versus CCL4).

To explore the potential mechanisms of the protective effect of astaxanthin in liver fibrosis, we firstly determined the expression of TGF-*β*1 in liver tissue, which is the most important cytokine contributing to the activation of HSCs. Astaxanthin (40 mg/kg and 80 mg/kg) significantly decreased the expression of TGF-*β*1 at the mRNA and protein levels (Figures 4(a) and 4(b)). In addition, we found smaller areas of TGF-*β*1 positive cells in the astaxanthin-treated group compared to the CCL4-treated group using immunohistochemical staining (Figure 4(c)), analyzed with Image-Pro Plus 6.0. Secondly, we confirmed that astaxanthin (40 mg/kg and 80 mg/kg) effectively increased the expression of MMP2, which participated in the regression of liver fibrosis through cleavage of the fibrillar ECM. Conversely, TIMP1, the major inhibitor of MMPs, was decreased by astaxanthin (80 mg/kg) (Figures 4(a) and 4(b)). These results showed that astaxanthin ameliorated liver fibrosis through decreased expression of TGF-*β*1 and a simultaneous stable MMP2/TIMP ratio.

### 3.4. Astaxanthin Inhibited the Expression and Translocation of NF-*κ*B in Liver Fibrosis

NF-*κ*B is one of the major nuclear factors and regulates multiple cytokines in the inflammatory response, including TGF-*β*1. Liver fibrosis is mainly due to stimulation of the chronic inflammatory response. Therefore, we determined the expression of NF-*κ*B in nuclei. As expected, the expression of NF-*κ*B was successfully inhibited by astaxanthin (80 mg/kg) (Figures 4(a) and 4(b)). NF-*κ*B positive cells were also reduced by astaxanthin (40 mg/kg and 80 mg/kg) with immunohistochemical staining. Hence, these results showed that astaxanthin might decrease the expression of TGF-*β*1 through downregulating NF-*κ*B signal pathway.

### 3.5. Astaxanthin Downregulated the Expression of LC3 and Beclin-1 and Decreased the Formation of Autophagosomes in Liver Fibrosis

**Figure 5:**
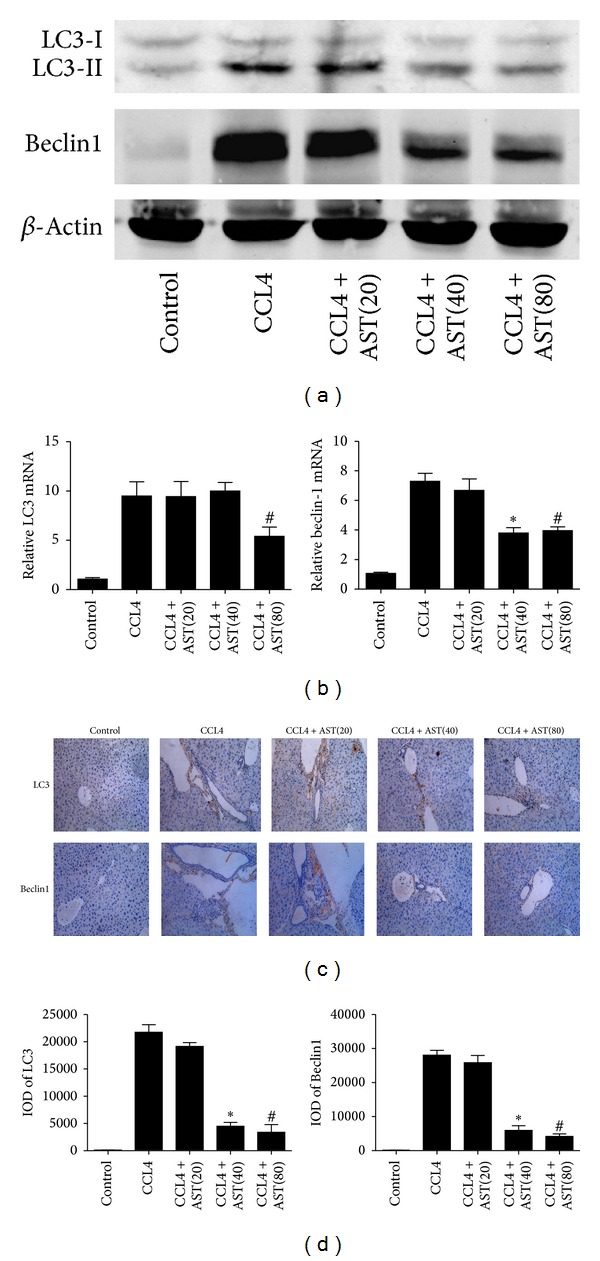
Effect of astaxanthin on the regulation of autophagy in HSCs. (a) The analysis of western blotting showed that astaxanthin (40 mg/kg and 80 mg/kg) obviously decreased the expression of LC3-II and beclin-1. (b) The mRNA levels of beclin-1 were decreased by astaxanthin (40 mg/kg and 80 mg/kg) compared to CCL4 group, and the level of LC3 was decreased only with the dose of 80 mg/kg. Data are expressed as mean ± SD (*n* = 7, *P < 0.05 for CCL4 + AST(40) versus CCL4, ^#^ *P* < 0.05 for CCL4 + AST(80) versus CCL4). (c) The areas of positive cells of LC3 and beclin-1, mainly expressed in HSCs as showed, were visibly diminished by astaxanthin (40 mg/kg and 80 mg/kg) (original magnification: ×200). (d) The IODs of LC3 and beclin-1 were analyzed by Image-pro Plus 6.0. There existed a significant decrease with astaxanthin (40 mg/kg and 80 mg/kg) treatment (*n* = 7, *P < 0.05 for CCL4 + AST(40) versus CCL4, ^#^ *P* < 0.05 for CCL4 + AST(80) versus CCL4).

**Figure 6:**
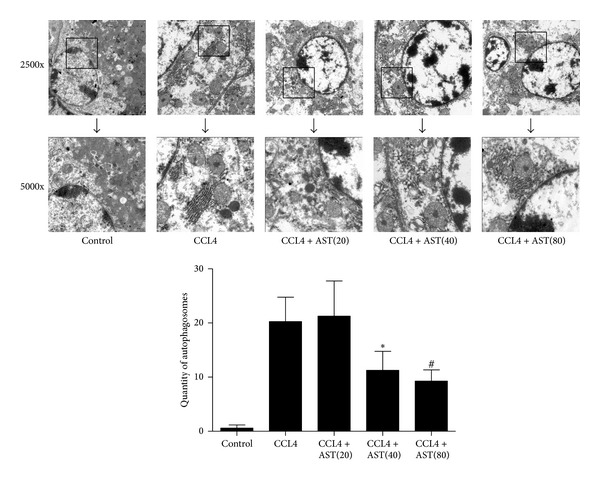
Effect of astaxanthin on the formation of autophagosome. As shown in picture, the amount of autophagosome obviously increased compared to control group. After astaxanthin (40 mg/kg and 80 mg/kg) treated, the amount of autophagosome significantly decreased, and the ultrastructure of cells exhibited more integrated (autophagosome was indicated with “→”) (original magnification: ×2500 and ×5000).

Autophagy can exacerbate liver fibrosis through the degradation of lipid droplets to provide energy. In this study, we determined the expression of LC3 and beclin-1 in all groups, which could indicate the level of autophagy. The expression of LC3 and beclin-1 decreased following astaxanthin (40 mg/kg and 80 mg/kg) administration both at the mRNA and protein levels (Figures 5(a) and 5(b). Immunohistochemical staining showed that LC3 and beclin-1 were mainly expressed in the cytoplasm of HSCs, and the areas of positive cells were significantly decreased by astaxanthin (40 mg/kg and 80 mg/kg) (Figure 5(c)), analyzed with Image-Pro Plus 6.0. In addition, we used electron microscopy to observe autophagosomes. Compared with the control group, increased lysosomes and autophagosomes were observed in the CCL4-treated group. As expected, following astaxanthin administration, cell structure showed more integrity, with fewer lysosomes and autophagosomes (Figure 6). These results indicated that astaxanthin might attenuate liver fibrosis through downregulation of autophagy in HSCs.

### 3.6. Astaxanthin Inhibited the Proliferation and Activation of HSC-T6 Cells

**Figure 7:**
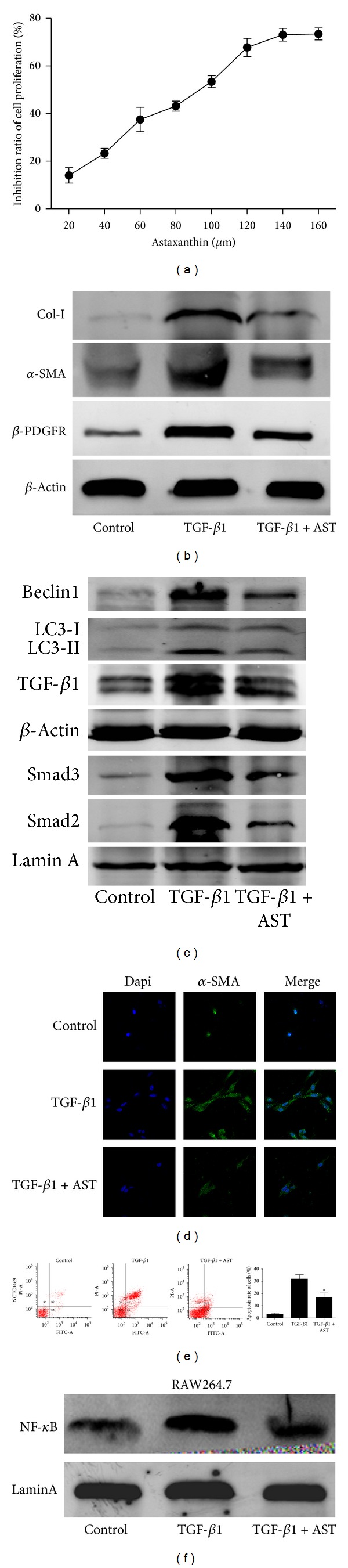
Effect of astaxanthin on HSC-T6, NCTC1469, and RAW264.7 cells. (a) The proliferation of HSC-T6 was detected by MTT. (b) The levels of *α*-SMA, *β*-pdfgr, and collagen I were detected by western blot. (c) The levels of beclin-1, LC3, TGF-*β*1, Smad2, and Smad3 were detected by western blot. (d) The expression of *α*-SMA in HSC-T6 cells was detected by immunofluorescence. (e) The apoptosis of NCTC1469 cells was detected by flow cytometry (*n* = 3, *P < 0.05 for TGF-*β*1 + AST(40) versus TGF-*β*1). (f) The result of western blot showed that astaxanthin decreased the expression of NF-*κ*B in RAW264.7 cells.

The HSC-T6 cells were treated with increasing concentrations of astaxanthin in an MTT assay. Results showed that astaxanthin caused a dose-depended reduction in HSC-T6 cells (Figure 7(a)). It indicated that astaxanthin could inhibit the proliferation of HSC-T6 cells. Here, we also calculated the IC_50_, which was used in our following experiment. On the other hand, astaxanthin (IC_50_) obviously decreased the expression of *α*-SMA, collagen I, and *β*-PDGFR with western blot (Figure 7(b)). The expression of *α*-SMA was also confirmed using immunofluorescence (Figure 7(d)). These results indicated that the activation of HSC-T6 was inhibited by astaxanthin (IC_50_).

### 3.7. Astaxanthin Downregulated the Expression of LC3 and Beclin-1 in HSC-T6

The levels of LC3 and beclin-1 were detected in HSC-T6 using western blot. Results showed that the expression of LC3 and beclin-1 was significantly decreased by astaxanthin (IC_50_) (Figure 7(c)). It indicated that astaxanthin could inhibit the level of autophagy in HSC-T6.

### 3.8. Astaxanthin Protected Liver Cells (NCTC1469) from Apoptosis and Inhibited the Level of NF-*κ*B in Macrophage (RAW264.7)

The result detected by flow cytometry showed that liver cells appeared a large percentage apoptosis after TGF-*β*1 administrated. However, the percentage of apoptosis was significantly decreased with astaxanthin treatment (Figure 7(e)). In addition, TGF-*β*1 was also administrated in RAW264.7. Results showed that the expression of NF-*κ*B was downregulated after astaxanthin treatment (Figure 7(f)).

## 4. Discussion

Antioxidants have been shown to have a protective effect on liver fibrosis both in animal models and clinical trials [25]. However, the effects of astaxanthin, which is a powerful and effective antioxidant, on liver fibrosis have not been investigated. Our study is the first to explore the effects of astaxanthin on liver fibrosis. Two models of liver fibrosis (CCL4 and BDL) were established in our experiments, which were identified as successful models of human liver fibrosis [26]. Our study confirmed that astaxanthin has a protective effect on liver fibrosis, as confirmed by the detection of biochemical indicators (ALT, AST, and hydroxyproline), and pathology (H&E, MT, and SR staining).

The study by Friedman confirmed that the activation of HSCs plays a pivotal role in liver fibrosis [9]. Further evidence has shown that HSCs are the principal collagen-producing cells and contribute to the deposition of ECM. Thus, reducing the number of activated HSCs may be a direct method of preventing liver fibrosis [27]. Here we found that the expression of *α*-SMA and *β*-PDGFR, markers of HSCs, decreased after astaxanthin treatment. In addition, the level of collagen I was downregulated, which is mainly expressed by HSCs. The same results were confirmed again in vitro in HSC-T6 cells. Therefore, these results show that HSCs were also the key target of astaxanthin similar to the majority of antifibrotic agents.

How does astaxanthin regulate the activation of HSCs in liver fibrosis? In chronic liver injury, injured and damaged cells such as hepatocytes and Kupffer cells release various profibrotic cytokines, for example, PDGF, TNF-*α*, and TGF-*β*1 [28]. During this process, these cytokines further stimulate the activation of HSCs [29]. Here, we found that astaxanthin effectively reduced the expression and release of TGF-*β*1. This may be the primary cause of the inhibition of HSCs activation by astaxanthin. It is well known that the NF-*κ*B family members are key regulators of inflammatory processes. In normal cells, NF-*κ*B is mainly located in the cytoplasm along with inhibitory proteins (I*Κ*b) and is translocated into the nucleus when activated by various stimuli. Recently, Friedman demonstrated that hepatic activation of NF-*κ*B signaling is sufficient to induce liver fibrosis [29]. Activation of the NF-*κ*B pathway results in the development of chronic inflammation, which in turn promotes liver fibrosis. Higher expression of NF-*κ*B in the nucleus has been confirmed in patients with chronic inflammation and liver fibrosis [30]. In addition, there have been many reports indicating that astaxanthin can inhibit the inflammatory response by downregulating NF-*κ*B [31, 32]. In the present study, our results showed that astaxanthin (80 mg/kg) decreased the expression of NF-*κ*B in the nucleus. Therefore, it is reasonable to believe that astaxanthin might decrease the expression of TGF-*β*1 by downregulating the level of NF-*κ*B in the nucleus. And we also found that astaxanthin could inhibit the expression of NF-*κ*B in macrophage through the experiments in vitro. In addition, TGF-*β*1/Smad2 and Smad3 signal pathway was also the main pathway which could be regulated in liver fibrosis. According to the results shown in Figure 4, we also could conclude that astaxanthin might partly ameliorate liver fibrosis through downregulating TGF-*β*1/Smad2 and Smad3 pathway.

MMPs, a family of zinc metalloendopeptidase, have the ability to activate HSCs in fibrosis, which are mainly from autocrine effects on HSCs. Conversely, MMPs might also contribute to degradation of the protein components of ECM and promote the apoptosis of activated HSCs. Therefore, MMPs play a dual role in liver fibrosis [33]. In our study, we found that astaxanthin increased the expression of MMP2 compared to the CCl4-treated group. Hence, we suspected that MMP2, which was increased by astaxanthin in our model, may cleave fibrillar ECM. TIMPs, which are inhibitors of metalloproteinases, are also highly expressed in liver fibrosis. It was reported that an imbalance in MMPs/TIMPs promoted the development of fibrosis. Our results showed that astaxanthin ameliorated liver fibrosis partly by preserving the balance between MMP2/TIMP1.

Under stress conditions, macroautophagy, which is commonly referred to as autophagy, is a programmed process involved in the degradation and recycling of cellular proteins, and removing damaged cell organelles. In most instances, autophagy has a beneficial role in maintaining cell homeostasis. However, excessive autophagy can also cause the degradation of necessary structure and damage in normal cells [34–37]. Recently, it was reported that autophagy exacerbated liver fibrosis through the degradation of lipid droplets in HSCs providing energy for the activation of HSCs [9]. Our results confirmed this phenomenon as the level of autophagy was upregulated in CCL4-induced liver fibrosis. Moreover, we found that astaxanthin downregulated the level of autophagy in HSCs, shown by reduced expression of LC3 and beclin-1 and a decrease in the number of autophagosomes. Our findings combined with those from previous reports indicate that astaxanthin may inhibit the activation of HSCs through downregulation of the level of autophagy in HSCs. The same results were also confirmed in vitro in HSC-T6 cells. Based on these results, we suggest that astaxanthin regulates the level of autophagy in HSCs. Recently, Kim et al. reported that TGF-*β*1 induced autophagy to suppress the accumulation of collagen I [38]. Similar results were obtained for TGF-*β*1 in mesangial cells and BME-UV1 cells [39, 40]. In our study, the levels of TGF-*β*1 and autophagy were simultaneously downregulated by astaxanthin. Hence, astaxanthin may downregulate autophagy by decreasing the expression of TGF-*β*1. Although previous reports have mainly demonstrated the protective effect of autophagy induced by TGF-*β*1, no contradictions among these studies were observed. Because autophagy has been confirmed to exacerbate liver fibrosis according to reports. Naturally, TGF-*β*1 is not the only factor involved in the regulation of autophagy. The regulatory mechanisms involved in autophagy in HSCs will be explored in our future work.

We also explored the effect of astaxanthin on liver cells and macrophage after TGF-*β*1 treatment. Results showed that astaxanthin could protect liver cells from apoptosis and inhibit the level of NF-*κ*B in macrophage. It indicated that astaxanthin had an effect on liver cells and macrophage in liver fibrosis models.

## 5. Conclusion

In conclusion, our study confirmed the following results: (1) astaxanthin ameliorated liver fibrosis (CCL4 and BDL). (2) Astaxanthin inhibited the activation of HSCs in vivo and in vitro. (3) Astaxanthin inhibited the activation of HSCs by decreasing the expression of NF-*κ*B and TGF-*β*1 and maintained the balance between MMPs and TIMPs. (4) Astaxanthin may influence the energy metabolism in HSCs by downregulating the level of autophagy in HSCs.

## Figures and Tables

**Table 1:** 

Gene		Primer Sequence (5′→3′)
*β*-actin	Forward	GGCTGTATTCCCCTCCATCG
	Reverse	CCAGTTGGTAACAATGCCATGT

NF-*κ*B	Forward	ATGGCAGACGATGATCCCTAC
	Reverse	CGGATCGAAATCCCCTCTGTT

LC3	Forward	GACCGCTGTAAGGAGGTGC
	Reverse	AGAAGCCGAAGGTTTCTTGGG

Beclin1	Forward	ATGGAGGGGTCTAAGGCGTC
	Reverse	TGGGCTGTGGTAAGTAATGGA

MMP2	Forward	GGACAAGTGGTCCGCGTAAA
	Reverse	CCGACCGTTGAACAGGAAGG

TIMP1	Forward	CGAGACCACCTTATACCAGCG
	Reverse	ATGACTGGGGTGTAGGCGTA

*α*-SMA	Forward	CCCAGACATCAGGGAGTAATGG
	Reverse	TCTATCGGATACTTCAGCGTCA

TGF-*β*1	Forward	CCACCTGCAAGACCATCGAC
	Reverse	CTGGCGAGCCTTAGTTTGGAC

*β*-pdgfr	Forward	TGCTGCACAGAGACTCCGTA
	Reverse	GATGAGCTTTCCAACTCGACTC

Col 1*α*1	Forward	CAATGGCACGGCTGTGTGCG
	Reverse	AGCACTCGCCCTCCCGTCTT

Col 1*α*2	Forward	CTCATACAGCCGCGCCCAGG
	Reverse	AGCAGGCGCATGAAGGCGAG
